# Postoperative inulin supplementation is associated with microbiota remodeling and improved colonic anastomotic healing in LPS-primed mice

**DOI:** 10.3389/fmicb.2026.1895439

**Published:** 2026-10-21

**Authors:** Yingye Shen, Junyan Zhao, He Gan, Lei Wu, Xinyao Hu, Jun Wang, Zezhou He, Feiyang Yao, Wenqiang Tao, Junqian Shang, Yong Chen, Chenyang Wang, Gefei Wang

**Affiliations:** 1 Department of General Surgery, Jinling Hospital, Affiliated Hospital of Medical School, Nanjing University, Nanjing, China; 2 School of Medicine, Southeast University, Nanjing, China; 3 Research Institute of General Surgery, Jinling Hospital, Nanjing Medical University, Nanjing, China; 4 Department of General Surgery, Jinling Clinical Medical College, Nanjing University of Chinese Medicine, Nanjing, Jiangsu, China; 5 National Engineering Research Center for Biotechnology, College of Biotechnology and Pharmaceutical Engineering, Nanjing Tech University, Nanjing, China; 6 Research Institute of General Surgery, Jinling Hospital, Affiliated Hospital of Medical School, Nanjing University, Nanjing, China

**Keywords:** anastomotic healing, dietary fiber, inflammatory- and repair-associated immune markers, inflammatory priming, tissue-associated microbiota

## Abstract

**Background:**

Anastomotic leakage is a major colorectal surgical complication that may be exacerbated by perioperative inflammatory stress. How transcriptomic profiles and tissue-adherent microbiota change under such stress, and whether postoperative inulin supplementation is associated with microbiota changes and improved healing after inflammatory priming, remain unclear.

**Methods:**

Two independent animal experiments were conducted. Experiment 1 compared control (CON) and LPS-primed mice using scheduled, repeated-cross-sectional sampling for microbiota profiling on POD0, 1, 3, 5, 7, and 14 and for RNA-seq on POD0, 1, 3, 5, and 7. Experiment 2 used the same murine colonic anastomosis model to evaluate postoperative exclusive enteral nutrition (EEN) with or without inulin supplementation (dietary fiber, DF) in a 2 × 2 factorial design comprising CON, CON-DF, LPS, and LPS-DF groups. Healing was assessed by survival, recovery scores, anastomotic burst pressure, histology, immunostaining, and short-chain-fatty-acid quantification. Within Experiment 2, a dedicated *n* = 10/group survival/longitudinal cohort was followed through POD5, and a separate terminal cohort provided six surviving mice/group for POD5 assessments; five microscopy fields were averaged within each mouse.

**Results:**

LPS priming impaired healing and induced niche-specific microbiota alterations with altered bacterial microspatial patterns at the suture line. DF supplementation was associated with improved recovery and anastomotic strength, reduced inflammatory infiltration, increased proliferation, decreased apoptosis, and altered inflammatory- and repair-associated immune markers. The planned LPS-DF versus LPS survival comparison in the POD5 four-group cohort was not significant after multiplicity correction. At POD5, DF was associated with lower Shannon diversity and an altered short-chain-fatty-acid profile, including higher acetate; the LPS × inulin interactions for these measures were not significant.

**Conclusion:**

Brief postoperative inulin-supplemented EEN improved anastomotic healing following inflammatory priming, concurrent with microbiota remodeling and short-chain-fatty-acid differences, including higher acetate. These concurrent changes do not establish microbiota- or SCFA-mediated causality.

## Graphical abstract

**Figure:**
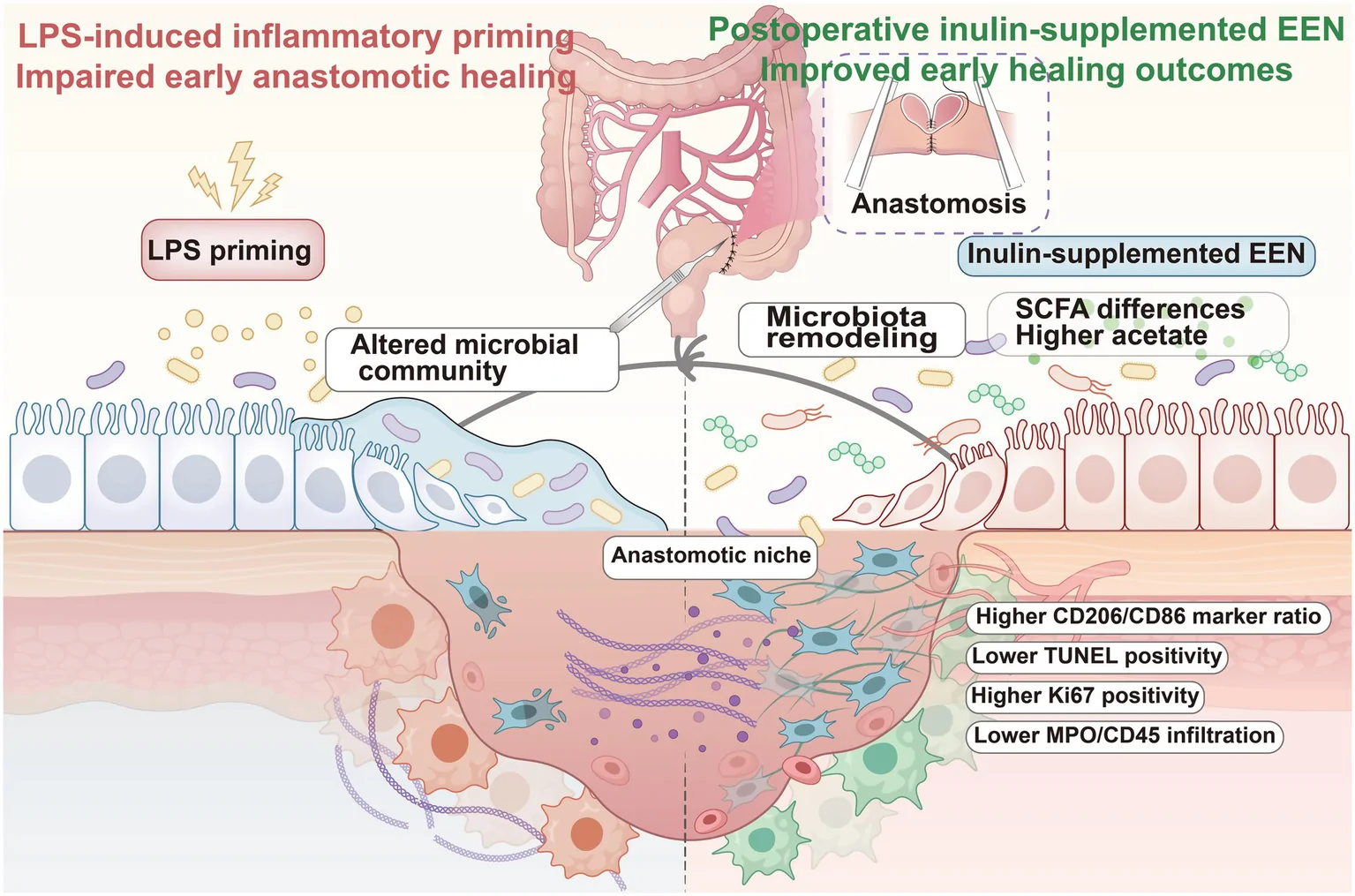
Acute LPS-induced inflammatory priming is associated with altered anastomotic niche ecology and impaired repair, whereas postoperative inulin-supplemented EEN is associated with microbiota remodeling, an altered SCFA profile including higher acetate, and improved early healing outcomes. The depicted relationships are associative and do not establish microbiota or SCFA mediation.

## 1 Introduction

Anastomotic leakage (AL) remains among the most devastating complications after colorectal surgery and occurs in approximately 5–19% of patients (Klein et al., 2012; Zhao et al., 2021; McDermott et al., 2015). AL substantially increases postoperative morbidity and mortality and is associated with an increased risk of local cancer recurrence (Bakker et al., 2014; Koedam et al., 2022). Although technical factors and severe ischemia have long been considered primary contributors (Jaferi et al., 2021), meticulous surgical technique alone cannot fully prevent anastomotic failure (Slieker et al., 2013). This limitation is particularly evident in high-risk settings characterized by preoperative systemic inflammation, such as that arising from perforation, obstruction, inflammatory bowel disease (IBD), or related endotoxemic stress (Sahami et al., 2016; Sartelli et al., 2014). These findings emphasize the importance of identifying biological factors affecting anastomotic healing beyond surgical techniques.

Acute endotoxemic inflammatory stress profoundly perturbs the anastomotic wound microenvironment. Systemic inflammation, together with local microcirculatory dysfunction, can compromise tissue perfusion and create an imbalance between oxygen delivery and metabolic demand in peri-anastomotic tissue (Strowitzki et al., 2021). Concurrently, infiltrating immune cells further increase the local metabolic burden. These stressors are accompanied by maladaptive transcriptional reprogramming within the mucosa and submucosa, which can hinder the timely resolution of inflammation and the initiation of reparative programs, including fibroblast activation and extracellular matrix (ECM) synthesis and remodeling (Sengul et al., 2025; van Praagh et al., 2021). Notably, anastomotic integrity is established within a narrow postoperative time window; failure to rapidly reconstitute an effective biological barrier and mechanical strength during this critical period predisposes the anastomosis to leakage.

Host repair responses are tightly intertwined with the gut microbiota, which has long been implicated in anastomotic healing outcomes (Cohn and Rives, 1955). Under homeostatic conditions, a protective wound milieu can sustain beneficial taxa, such as *Akkermansia muciniphila*, that support mucosal repair, in part through redox-related pathways (Alam et al., 2016). In contrast, perioperative perturbations, including bowel preparation, infection, and tissue injury, may destabilize the mucosa–microbe interface, driving the system towards a “leak-prone” ecological state (Shogan et al., 2014). Importantly, emerging evidence suggests that the risk is not determined by the community composition alone. Niche specificity, including tissue-associated versus luminal communities, and microspatial organization at the anastomotic site shape host–microbe interactions and downstream repair trajectories. In particular, the expansion of opportunistic pathobionts such as Enterococcus and Escherichia–Shigella is associated with increased matrix degradation, impaired collagen integrity, and compromised mechanical stability (Shogan et al., 2015). However, how endotoxaemic stress reshapes microbial succession across distinct intestinal niches and whether it promotes microspatial clustering at the anastomosis during the early healing window remain insufficiently defined (Alverdy et al., 2017).

Current clinical strategies to mitigate the AL risk, such as broad-spectrum antibiotics and systemic anti-inflammatory therapies, are limited by adverse effects and can further disrupt microbial ecology (Hakkarainen et al., 2015; Guyton and Alverdy, 2016). In contrast, microecological interventions, particularly nutritional approaches, offer a relatively low-risk means of modulating the postoperative environment (Lloyd et al., 2006; Tang et al., 2020). Dietary fiber (DF) promotes beneficial microbial networks and increases the production of short-chain fatty acids (SCFAs), notably butyrate, which reinforces epithelial barrier function and may facilitate immune resolution and tissue repair (Kok et al., 2021; Kastl et al., 2022; Hyoju et al., 2020).

Despite accumulating evidence of links among endotoxaemia, dysbiosis, and AL, critical gaps remain regarding the temporal and niche-specific dynamics of microbiota remodeling, particularly differences between the anastomotic tissue and luminal contents, and the emergence of microspatial clustering at the suture line. Moreover, how these ecological dynamics interface with early wound-healing processes—including transcriptomic programs, inflammation resolution, inflammatory- and repair-associated immune-marker profiles, and matrix maturation—remains unclear.

We addressed these questions using a model of acute LPS-induced systemic inflammatory priming. Across two independent experiments, a CON-versus-LPS repeated-cross-sectional time course was used to characterize niche-resolved microbiota and transcriptomic changes, and a separate four-group factorial experiment was used to examine whether postoperative DF supplementation was associated with microbiota remodeling and improved early anastomotic healing. The study was designed to characterize these concurrent microbial and healing outcomes rather than to establish microbiota- or metabolite-mediated causality.

## 2 Materials and methods

### 2.1 Animals and ethics statement

The animal procedures were approved by the Institutional Animal Care and Use Committee of the Drug Safety Evaluation Center of Jiangsu Province (SYXK (SU) 20210040) and performed in strict accordance with the institutional guidelines of Nanjing University. Female C57BL/6 J mice (8 weeks old, weighing 18–20 g) were purchased from Jiangsu Qinglongshan Biotechnology Co., Ltd. All the mice were housed in an SPF facility at 22 ± 2 °C under a 12-h light/dark cycle and provided ad libitum access to a standard rodent diet and water. The animals were acclimated to the laboratory environment for at least 7 days prior to any experimental procedures. Females were selected for consistency with the pilot experiments and related murine work and to reduce intermale aggression; the female-only design limits generalizability to males.

### 2.2 Experimental design and grouping

The study consisted of two independent animal experiments. Experiment 1 compared CON (saline) and LPS mice and used scheduled sampling on POD0, 1, 3, 5, 7, or 14; different animals were sampled at each time point, so these data represent repeated cross-sectional observations rather than repeated measurements from the same mice. Microbiota profiling was performed on POD0, 1, 3, 5, 7, and 14, whereas RNA sequencing was performed only on POD0, 1, 3, 5, and 7. Experiment 2 used a 2 × 2 factorial design to evaluate preoperative LPS priming and postoperative nutritional intervention, comprising CON, LPS, CON-DF (saline + DF), and LPS-DF (LPS + DF) groups. The EEN regimen consisted of a fibre-free short-peptide enteral formula (Nutrison; Enteral Nutritional Suspension, SP; Nutricia); its composition is provided in Supplementary Table S1. DF supplementation was achieved by adding inulin (Shanghai Yuanye Bio-Technology; S11143) at 10% (w/v), and DF therefore denotes the inulin-supplemented EEN regimen throughout this study. Individual formula intake was not recorded and pair-feeding or viscosity−/osmolality-matched controls were not used; therefore, the observed effects cannot be attributed solely to inulin or fibre fermentation. Animals were group-housed at 4–5 mice per cage, and the two experiments were analyzed separately and were not pooled.

Sample sizes were determined on the basis of prior experience and experimental feasibility; no formal *a priori* power calculation was performed. Experiment 1 included independent scheduled-harvest animals at each time point, with complete animal flow and assay-specific biological sample sizes provided in Supplementary Table S5. Experiment 2 included a dedicated four-group survival/longitudinal cohort of 10 mice per group followed through POD5. A separate POD5 terminal cohort was enrolled sequentially to obtain six evaluable POD5 survivors per group: 7 CON, 7 CON-DF, 13 LPS, and 8 LPS-DF mice underwent surgery, and 1, 1, 7, and 2 mice, respectively, died or reached a humane endpoint before POD5, leaving six evaluable animals per group for ABP, histological, and immunofluorescence analyses. Accordingly, these POD5 terminal outcomes represent survivor analyses. No animals or data were shared between Experiments 1 and 2.

### 2.3 Preoperative preparation and surgical procedures

The mice received a sublethal intraperitoneal injection of 2 mg/kg LPS or sterile saline 24 h prior to surgery to induce systemic endotoxaemia. During this period, the mice were fasted with unrestricted access to water. For perioperative analgesia, meloxicam (Sigma–Aldrich, PHR17995, 5 mg/kg) was administered subcutaneously 30 min before the procedure. Anesthesia was maintained via inhaled isoflurane, and the core body temperature was monitored rectally and maintained at a stable range using a heating pad.

Mice were assigned using a random-number table, allocation was concealed until assignment, and surgical order was interleaved across groups. Briefly, a 1.5-cm midline laparotomy was conducted under sterile conditions. The distal colon was transected and reanastomosed in an end-to-end manner using interrupted 9–0 nylon sutures under a microscope. After verifying the integrity of the anastomosis with a saline leak test, the abdominal wall was closed in two layers with 5–0 Vicryl and nylon sutures. All procedures were performed by the same surgeon. Outcome assessors, image selectors, and image quantifiers were blinded to coded group labels, and POD5 samples were processed in one batch.

### 2.4 Postoperative care

Following surgery, the mice received a subcutaneous injection of sterile saline (0.5 mL) for fluid resuscitation and meloxicam (5 mg/kg) for analgesia. Meloxicam was administered once daily for 48 h postoperatively. In Experiment 1, EEN was provided through POD7. In Experiment 2, EEN was provided through POD5. Feeding bottles and inulin-containing formula were replaced at the same time each day, but intake was not recorded; therefore, actual inulin exposure (g/kg/day), energy intake, and macronutrient intake could not be estimated. Pair-feeding and viscosity- or osmolality-matched controls were not used. In the dedicated four-group *n* = 10/group cohort, body weight, survival status, and Murine Sepsis Score were recorded through POD5. Death or attainment of a predefined humane endpoint was treated as an event; animals alive at POD5 were administratively censored.

### 2.5 Sample collection and processing protocols

At the designated time points, the mice were euthanized with an overdose of sodium pentobarbital (Sigma–Aldrich, 50002, 80 mg/kg). Whole blood was harvested via terminal cardiac puncture, and serum was subsequently isolated by centrifugation (3,000 × g for 15 min at 4 °C) and stored at −80 °C.

For mechanical testing, an intact colonic segment encompassing the anastomosis was harvested for immediate anastomotic burst pressure (ABP) testing. The 2-cm anastomotic segment was then divided: one portion was snap-frozen in liquid nitrogen and stored at −80 °C for subsequent transcriptomic (RNA-seq) and proteomic analyses, while the remaining portion was fixed with 4% paraformaldehyde for the histological evaluation.

For SEM, anastomotic samples were fixed with 2.5% glutaraldehyde (Sigma–Aldrich, 340855) at 4 °C for 24 h. For microbial profiling, anastomotic tissues as well as the colonic and cecal contents were collected aseptically and stored at −80 °C for 16S rRNA sequencing. Before tissue-associated microbial sampling, specimens were rinsed with sterile PBS and collected with sterile instruments. Extraction blanks and PCR no-template controls were not included, and no dedicated contaminant-identification/filtering procedure was applied.

### 2.6 Functional and histological assessments

#### 2.6.1 Macroscopic and functional evaluations

Two independent investigators who were blinded to the treatment groups assessed macroscopic healing at the time of sacrifice. The anastomotic complication score (ACS) was utilized to quantify intra-abdominal adhesions and identify leakage, with the latter defined by the presence of faecal peritonitis, gross extravasation, or localized abscesses (Supplementary Table S3) (Weber et al., 2023; Miltschitzky et al., 2021).

The perianastomotic adhesion area was quantified using standardized necropsy photographs captured at a fixed focal distance with a scale bar. Using ImageJ software (National Institutes of Health, United states), adhesions within a predefined perianastomotic region centered on the anastomosis were manually traced, and the total area was calculated in cm^2^.

On PODs 3, 5, and 7, mechanical strength was assessed using a 4-cm colonic segment centered on the anastomosis. The segment was connected to a medical syringe pump (Shanghai Lande Medical Equipment Co., Ltd., China) and a digital pressure gauge (SUX-YB80; Suxun, China; range 0–50 kPa; accuracy class 0.2). Normal saline was infused at 2 mL/min. The gauge was automatically zeroed to atmospheric pressure before each measurement; no independent external calibration was performed. ABP was defined as the peak pressure immediately before visible leakage or rupture with a pressure drop. Failure location was recorded as the anastomotic site, and the assessor was blinded to group allocation. Additionally, the lengths of the proximal and distal colon were measured as surrogate indicators of intestinal inflammation.

#### 2.6.2 Histopathological and immunohistochemical analyses

Tissue sections (4 μm) were prepared and stained with hematoxylin and eosin (H&E) and Masson’s trichrome (MT). A blinded semiquantitative histological scoring system was used to assess inflammatory cell infiltration, granulation tissue development, angiogenesis, and collagen deposition (Weber et al., 2023; Miltschitzky et al., 2021; Phillips et al., 1992).

For MT quantification, the region of interest (ROI) in the wound bed was defined as the granulation tissue area. Collagen deposition was quantified as the percentage of collagen-positive area within this ROI using automated color deconvolution and thresholding in ImageJ. The parameters remained consistent across all the samples.

For IHC and multiplex immunofluorescence staining, primary antibodies against markers of immune cell infiltration and macrophage-associated inflammatory and repair markers were used. Immunohistochemistry (IHC) was performed using antibodies against MPO (1:200, Abcam, ab208670) and CD45 (1:200, Abcam, ab10558). Immunofluorescence (IF) staining was performed using antibodies against F4/80 (1:200, Abcam, ab300421), CD86 (1:200, Abcam, ab220188), CD206 (1:200, Abcam, ab64693), Ki67 (1:200, Abcam, ab16667), and DAPI (Sigma–Aldrich, D9542). TUNEL staining (Abbkine, KTA2011) was performed to identify apoptotic cells within the anastomotic site according to the manufacturer’s protocol. A quantitative analysis of representative ROIs identified by a pathologist was performed using ImageJ software (National Institutes of Health, United States). The markers were quantified by calculating the percentage of the positive area, the mean fluorescence intensity (MFI), or the number of positive cells per high-power field. The results for proliferation and apoptosis are presented as the percentage of positive cells relative to the total number of nuclei. All assessments were conducted in a blinded manner to ensure objectivity. One section per mouse was analyzed; five non-overlapping fields were quantified and averaged within each mouse before analysis. The mouse, rather than the field, was the biological unit, and image selection and quantification were performed under blinded group codes.

#### 2.6.3 Visualization of bacterial aggregates and microbial topography

The spatial distribution of the microbiota at the anastomotic site was visualized using SEM and FISH. For SEM, samples were fixed with 2.5% glutaraldehyde, dehydrated through a graded ethanol series, and subjected to critical-point drying before imaging with a Hitachi SU8010 microscope.

For FISH, paraffin-embedded sections were hybridized using the universal bacterial probe EUB338 (labelled with Cy3, Sigma–Aldrich, MBD0033) to visualize bacterial positioning relative to the sutures and the wound interface. The bacterial density was quantified by counting the number of EUB338-positive bacterial cells normalized to the number of DAPI-stained nuclei within the anastomotic granulation tissue ROI (EUB338^+^ counts/DAPI^+^ nuclei), providing a relative bacteria-to-host spatial metric at the host–microbe interface. This ratio was not interpreted as absolute bacterial load. EUB338 FISH and SEM demonstrate localization or aggregation but do not establish taxonomic identity, viability, extracellular-matrix production, or bona fide biofilm architecture.

### 2.7 Molecular and microbiota analyses

#### 2.7.1 16S rRNA sequencing and analysis

Microbial DNA was extracted from luminal contents and tissue-associated samples using the HiPure Soil/Stool DNA Kit (Magen, China, D314401). The V3–V4 region of the 16S rRNA gene was amplified using the primers 341F and 806R and sequenced on the Illumina NovaSeq 6000 platform. Raw reads were quality-filtered (FASTP, v0.18.0), and sequences were clustered into operational taxonomic units (OTUs) at 97% similarity using UPARSE. Chimaeras were removed using UCHIME. Effective tags were used to determine the abundance statistics of the OTUs. Representative sequences were annotated using SILVA (v138.2). Alpha diversity was assessed using the Shannon index. Beta diversity was calculated based on Bray–Curtis dissimilarity and visualized by performing principal coordinate analysis (PCoA), and group differences were assessed using PERMANOVA (adonis, 999 permutations). Differentially abundant taxa were identified using LEfSe with an LDA score >3.0 and *p* < 0.05. The functional profiles were inferred using PICRUSt2 and annotated to KEGG pathways. The functional capacity was inferred using PICRUSt2 and should be interpreted as the predicted potential rather than the measured activity. Heatmaps were generated after row-wise z-score normalization. The Experiment 1 CON-versus-LPS time series and the Experiment 2 POD5 four-group cohort were sequenced in separate batches and analyzed separately. In the time-series batch, raw reads ranged from 31,331 to 102,826, effective tags from 28,345 to 84,785, and retention from 74.67 to 92.79%; the corresponding four-group ranges were 52,350–125,465, 49,828–122,977, and 82.05–98.47%. Within each batch, libraries were rarefied to the minimum effective-tag count (28,345 and 49,828, respectively) before diversity analysis. The original 97%-OTU workflow was retained for consistency, and no ASV reanalysis was performed. Sample-level 16S sequencing and preprocessing metrics are provided in Supplementary Table S10. No extraction/PCR negative controls or dedicated contaminant filtering were available; LEfSe and PICRUSt2 results are therefore treated as exploratory, and PICRUSt2 denotes predicted functional potential only.

At the designated sampling time, 100 mg (wet weight) of colonic contents was collected from each mouse, snap-frozen in liquid nitrogen, and stored at −80 °C without repeated freeze–thaw cycles. Samples were pretreated with 25% (w/v) metaphosphoric acid, centrifuged, and membrane-filtered. Butyrate and acetate were quantified using an external-standard HPLC method on an Agilent 1260 Infinity II system with a variable-wavelength detector and Hi-Plex H column (300 × 7.7 mm, 8 μm) at 60 °C. The mobile phase was 5 mmol/L sulfuric acid at 0.7 mL/min; the injection volume was 20 μL and detection was at 210 nm. Standards for butyrate (n-butyric acid, 99.5%) and acetate (acetic acid, 99.8%) were obtained from Shanghai Macklin Biochemical Co., Ltd. No derivatization or internal standard was used. Acetate and butyrate concentrations were expressed in mmol/L.

#### 2.7.2 Transcriptomic and protein expression analyses

Total RNA was extracted using TRIzol reagent (Sigma–Aldrich, T3934). POD0 was defined as tissue collected immediately after completion of surgery, and CT and LT denote control (CON) tissue and lipopolysaccharide-treated (LPS) tissue, respectively. At least three biological replicates were planned for each group–time cell at POD0, 1, 3, 5, and 7; additional samples were submitted to account for potential library-construction failure, and all libraries that passed quality control were retained, resulting in 3–5 samples per group–time cell. All libraries were prepared and sequenced in a single analytical batch on an Illumina NovaSeq 6000 platform. Across samples, raw sequencing depth ranged from 36.25 to 43.37 million reads, clean-read retention from 99.34 to 99.55%, Q30 from 93.64 to 94.42%, and total mapping rate from 97.57 to 98.18%. Reads were aligned to the *Mus musculus* GRCm39 reference genome with Ensembl release 112 annotation using HISAT2 v2.1.0; StringTie v1.3.4, RSEM v1.2.19, and DESeq2 v1.20.0 were used for transcript reconstruction, abundance estimation, and differential-expression analysis. DEGs were defined using |log₂ fold change| > 1 and adjusted *p* < 0.05. RNA quality was assessed using NanoDrop spectrophotometry and gel electrophoresis. Actual sample sizes and sample-level sequencing quality metrics are provided in Supplementary Table S11. Functional enrichment used KEGG and GO databases to identify overrepresented biological categories.

Transcriptomic findings were validated by quantitative PCR (qPCR) conducted using an ABI 7500 Real Time PCR System. Total RNA was isolated from colonic tissues with the MiniBEST Universal RNA Extraction Kit (TaKaRa; 9767) and quantified using a NanoDrop 2000 spectrophotometer. For mRNA analysis, cDNA was synthesized using PrimeScript RT Master Mix (TaKaRa; RR430S). The qPCR assays were performed in technical triplicate using TB Green Premix Ex Taq II (TaKaRa; RR820L). The relative expression levels of target genes were calculated via the 2^−ΔΔCt^ method and normalized to the expression of Gapdh as the internal control. All primer sequences are provided in Supplementary Table S4.

Anastomotic tissue samples (50 mg) were homogenized in 0.5 mL of ice-cold RIPA buffer (Sigma–Aldrich, V900854) supplemented with a complete protease inhibitor cocktail. Following homogenization, the lysate was maintained on ice for 45 min to facilitate complete lysis. The mixture was subsequently centrifuged at 4 °C for 15 min (13,000 × g) to pellet the cellular debris, after which the protein-rich supernatant was collected. The resulting protein-containing supernatant was aliquoted and cryopreserved at −80 °C for further analysis. Protein concentrations were quantified using a bicinchoninic acid (BCA, Sigma–Aldrich, 71285-M) assay kit. Proteins were resolved by electrophoresis on 6–10% SDS-polyacrylamide gels and subsequently transferred to PVDF membranes (0.45 μm, Millipore; IPVH00010). After transfer, the membranes were blocked with 5% nonfat dry milk at room temperature and then incubated overnight at 4 °C with the following primary antibodies from Abcam: anti-iNOS (1:1,000; ab178945), anti-Arg1 (1:1,000; ab124917), anti-TGF-β1 (1:1,000; ab179695), anti-PI3K p85 (1:1,000; ab191606), anti-total Akt (1:1,000; ab8805), anti-phospho-Akt (Ser473) (1:1,000; ab81283), anti-Bax (1:1,000; ab32503), anti-Bcl-2 (1:1,000; ab182858), and anti-β-actin (1:5,000; ab6276). After the membranes were washed with Tris-buffered saline with Tween 20 (TBST), HRP-conjugated secondary antibodies (anti-rabbit, Abbkine, A21020 and anti-mouse, Abbkine, A21010) were applied. The expression levels of the proteins are presented as percentages (%) relative to *β*-actin expression, with the p-Akt/total Akt ratio reported as an associative protein-level readout. The band intensities were quantified using ImageJ software.

### 2.8 Statistical analyses

Routine cross-sectional analyses and figure generation were performed in OriginPro 2023b. Four-group POD5 outcomes from Experiment 2 were analyzed using 2 × 2 factorial linear models containing terms for LPS, inulin, and the LPS × inulin interaction; the Bcl-2/Bax ratio was analyzed on the natural-log scale. Main effects, interactions, F-statistics, denominator degrees of freedom, exact two-sided *p* values, effect estimates, and 95% CIs are reported in Supplementary Tables S6–S7, with Tukey-adjusted pairwise comparisons where appropriate. Body weight and Murine Sepsis Score were analyzed using random-intercept mixed-effects models with categorical time and fixed effects for LPS × inulin × time. Measurements at or after death/humane endpoint were treated as missing and were not imputed; survivor-only sensitivity analyses were also conducted. The four-group survival analysis in Experiment 2 through POD5 used Kaplan–Meier estimates and the log-rank test. Four prespecified simple-effect contrasts were adjusted by the Holm method, and pair-specific Cox models were used to calculate exploratory HRs and 95% CIs. Full survival data and numbers at risk are provided in Supplementary Table S8, and mixed-effects/sensitivity outputs in Supplementary Table S9. Reanalysis was performed using Python 3.12.13, pandas 2.2.3, NumPy 2.3.5, and SciPy 1.17.0. A two-sided *p* < 0.05 was considered significant.

## 3 Results

### 3.1 Preoperative LPS treatment impairs postoperative recovery and reduces the mechanical strength of colonic anastomoses

We first monitored postoperative recovery following LPS challenge to evaluate the systemic effect of preoperative endotoxaemia. Mice subjected to LPS-induced systemic inflammatory priming exhibited immediate systemic deterioration, characterized by rapid weight loss, which reached a maximum reduction of 21.65 ± 1.90% on POD 3 (*p* < 0.001; Figure 1B). This weight loss was accompanied by significantly elevated Murine Sepsis Scores through POD 4 (*p* < 0.01; Figure 1C).

**Figure 1:**
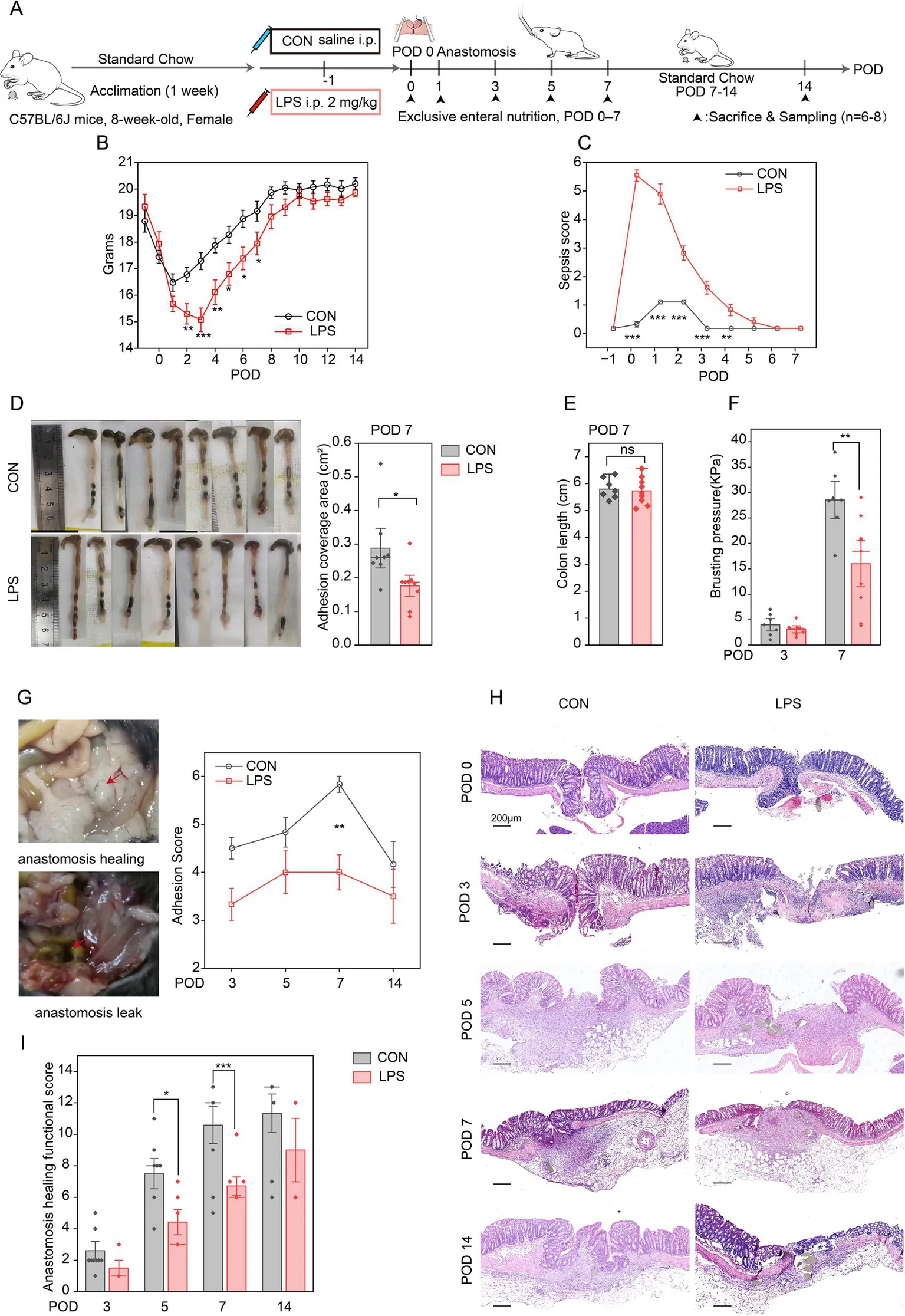
Experiment 1: LPS-induced inflammatory priming is associated with impaired postoperative recovery and compromised colonic anastomotic healing. **(A)** Experimental scheme: female C57BL/6 J mice received saline or LPS (2 mg/kg, i.p.) 24 h before colonic anastomosis; exclusive enteral nutrition was provided from POD0 to POD7, and scheduled terminal sampling was performed on POD0, 1, 3, 5, 7, or 14. This scheduled-harvest time-course experiment was independent of the four-group intervention experiment presented in Figures 6–8. **(B)** Postoperative body-weight trajectories. **(C)** Clinical sepsis scores following LPS challenge and surgery. **(D)** Representative gross images of perianastomotic protective adhesions on POD7 and quantification of the adhesion coverage area (cm^2^). **(E)** Colon length on POD7. **(F)** Anastomotic bursting pressure (ABP) on POD3 and POD7. **(G)** Representative intra-abdominal images and adhesion scores from POD3 to POD14. **(H)** Representative H&E-stained sections of the anastomosis from POD0 to POD14 (scale bar, 200 μm). **(I)** Anastomotic functional healing scores from POD3 to POD14. Data are presented as mean ± SEM with individual animals shown; each dot represents one animal. Statistical comparisons were performed as indicated in the panels.

We assessed perianastomotic adhesions, which contribute to the biological sealing of the anastomotic region, to determine whether the systemic deterioration was associated with impaired local wound healing. Macroscopically, LPS-primed mice displayed significantly reduced perianastomotic adhesion coverage (0.17 ± 0.02 cm2 vs. 0.29 ± 0.03 cm^2^ in the CON group; Figure 1D) and lower adhesion scores (Weber et al., 2023) at POD 7 (*p* < 0.01; Figure 1G). On POD 7, the colon length was comparable between the CON and LPS groups (ns; Figure 1E). The anastomotic burst pressure (ABP) was measured as the primary endpoint of mechanical strength to quantify the functional integrity of the anastomosis. While ABP was comparable between groups at POD 3, the LPS group showed a 43.98% reduction by POD 7 (16.00 ± 3.02 kPa vs. 28.56 ± 2.40 kPa, *p* < 0.01; Figure 1F).

Throughout the histological time course, epithelial continuity and structural maturation at the anastomotic site progressed in the control mice but remained delayed in mice with endotoxaemia (Figure 1H), with reduced mucosal continuity during POD 3–POD 7 and persistent remodeling-related abnormalities observed by POD 14. Consistently, anastomosis healing functional scores(Weber et al., 2023) were comparable at POD 3 but were significantly lower in the LPS group at POD 5 (*p* < 0.05) and POD 7 (*p* < 0.001); the scores increased by POD 14 but remained lower than those of the controls (*p* < 0.05) (Figure 1I). Collectively, these findings indicate that endotoxaemia impairs local protective adhesions and mechanical integrity, consistent with increased vulnerability to anastomotic failure.

### 3.2 LPS-induced inflammatory priming is associated with disrupted niche-specific microbial succession and an altered microspatial organization at the anastomosis

Given the established links between the microbiota and mucosal repair, we next investigated whether the observed impaired healing was associated with niche-specific microbial dysbiosis. We characterized how preoperative endotoxaemia affects postoperative microbial dynamics by profiling microbial communities from three anatomical sites across a 14-day post-operative course.

Principal coordinate analysis (PCoA) based on the Bray–Curtis distances showed that microbial communities clustered primarily by anatomical site (PERMANOVA: *p* = 0.001, *R*^2^ = 0.291), highlighting a strong niche-specific effect (Figure 2B). Within each site, the postoperative trajectories shifted in a time-dependent manner from POD 0 to POD 14 in all groups; however, the trajectories in the LPS group deviated significantly from those in the CON group. Across all three niches, the Bray–Curtis dissimilarity increased early after surgery but then gradually decreased, with the greatest separation observed between POD 0 and POD 5 (Figures 2C–E), indicating a profound disruption of microbial succession in mice with endotoxaemia. Because this global analysis combined anatomical site, condition, and time and was dominated by site, it is interpreted descriptively rather than as a treatment-specific LPS effect.

**Figure 2:**
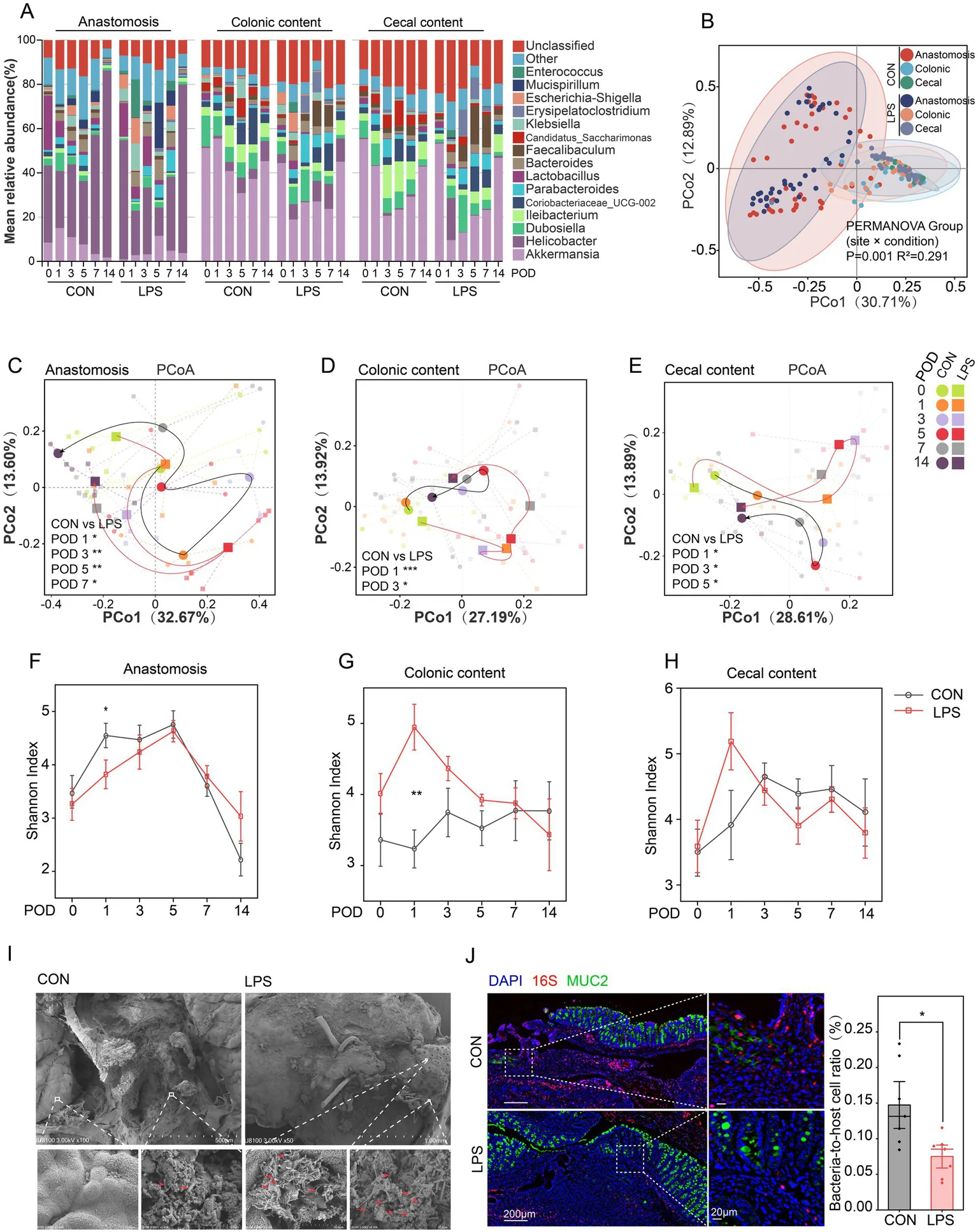
Endotoxemic stress disrupts niche-specific microbial succession and microspatial organization at the colonic anastomosis. **(A)** Genus-level community composition across postoperative time (POD 0, 1, 3, 5, 7, and 14) in anastomotic tissue, colonic content, and cecal content from control (CON) and LPS-challenged mice, shown as mean relative abundance. **(B)** Principal coordinate analysis (PCoA) of Bray–Curtis dissimilarities for all samples; ellipses denote the six site-by-condition groups (anastomosis/colonic/cecal × CON/LPS); overall group effect was tested by PERMANOVA (Bray–Curtis), *R*^2^ = 0.291, *p* = 0.001. **(C–E)** Site-stratified PCoA for anastomotic tissue **(C)**, colonic content **(D)**, and cecal content **(E)**, with points colored by POD and trajectories illustrating postoperative succession within each group; same-day CON vs. LPS differences were assessed by PERMANOVA (Bray–Curtis) at each POD, and significant PODs are indicated in-panel. **(F–H)** Alpha diversity (Shannon index) over time in anastomotic tissue **(F)**, colonic content **(G)**, and cecal content **(H)**. **(I)** Scanning electron microscopy (SEM) of the anastomotic surface showing microspatial bacterial organization in the CON and LPS groups (representative images). **(J)** Fluorescence *in situ* hybridization (FISH) of the anastomotic region stained with DAPI (nuclei), a universal bacterial 16S probe, and MUC2 (mucus), shown at low and high magnification, with quantification of the relative bacteria-to-host spatial ratio; this metric was not interpreted as absolute bacterial load. Data are presented as mean ± SEM where applicable. *p* < 0.05, *p* < 0.01, *p* < 0.001 (as indicated). Scale bars are shown in the panels.

Interestingly, we observed divergent alpha diversity patterns across niches. Notably, the alpha diversity in the anastomotic tissue increased from POD 0 to POD 5, whereas both the colonic and cecal luminal contents showed a transient increase in alpha diversity on POD 1, followed by a partial decrease thereafter, which was consistent with a perturbed niche-specific assembly process at the injury site (Figures 2F–H).

Microspatial imaging using SEM and FISH provided additional qualitative and quantitative insights into bacterial organization. Although the anastomoses of control mice maintained an organized mucosal surface, those from LPS-challenged mice exhibited dense bacterial clustering along the sutures and necrotic debris (Figure 2I). FISH quantification based on EUB338^+^ signal counts normalized to DAPI^+^ nuclei within the granulation-tissue ROI revealed a lower bacteria-to-host spatial ratio in endotoxemic tissues (*p* < 0.05; Figure 2J). These findings suggest that endotoxaemia is associated with microspatial redistribution and focal bacterial aggregation rather than a uniform increase in the relative EUB338-to-DAPI spatial ratio.

Taxonomic profiling (Figure 2A) indicated that the LPS challenge was associated with higher relative abundances of pathobionts, including Enterococcus and Escherichia–Shigella, particularly during PODs 1–3. LEfSe and cladogram analyses further revealed enrichment of Enterococcaceae and Enterobacteriaceae in the endotoxaemia group, whereas Akkermansiaceae and Bacteroidaceae remained relatively stable in the CON group (Figure 3A). Consistent with these findings, the relative abundance of Enterococcus exhibited an early peak on POD 1 (Figure 3B), whereas the mucoprotective commensal Akkermansia showed lower relative abundance during the critical early repair window.

**Figure 3:**
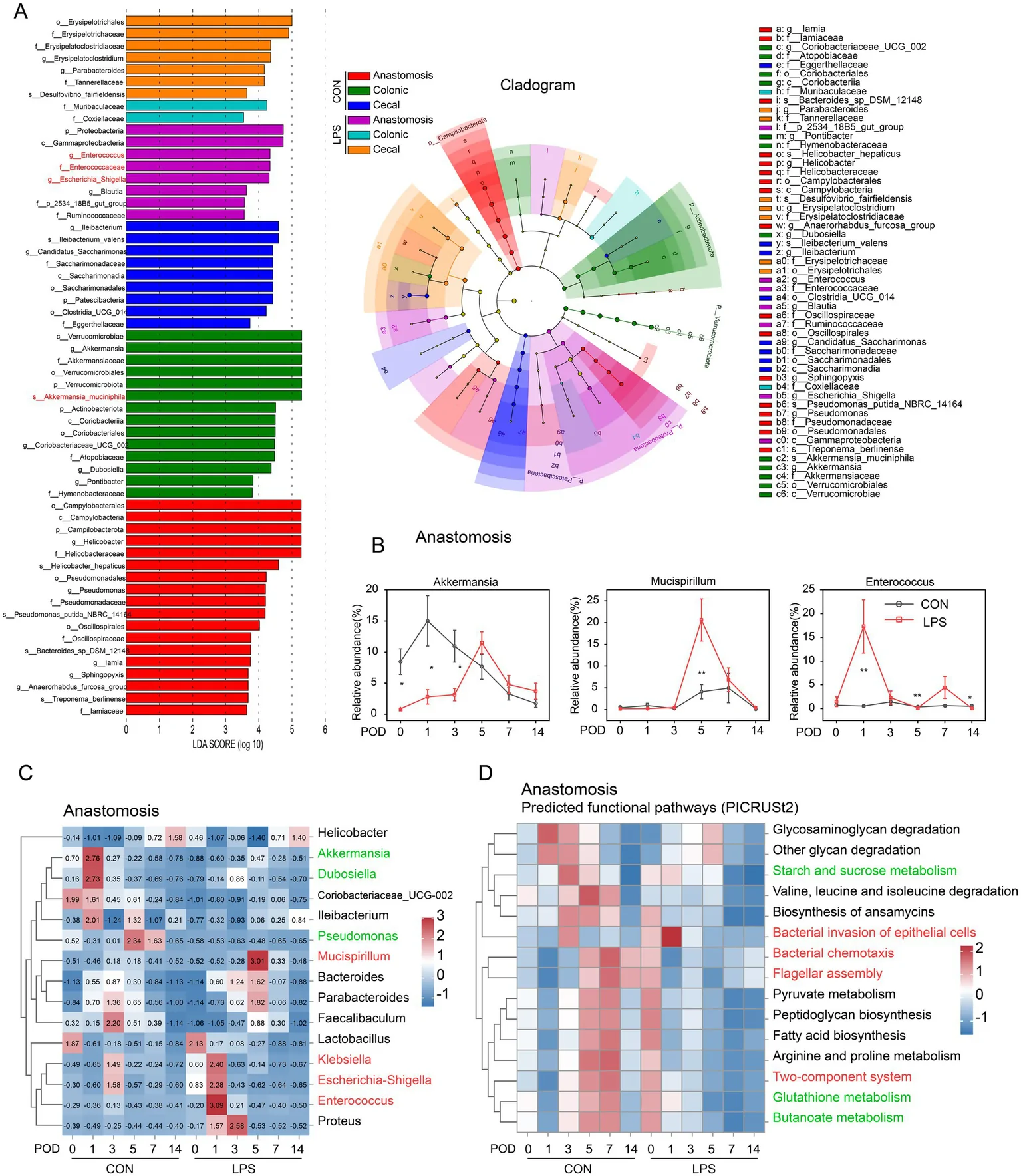
Endotoxemic stress reshapes anastomosis-associated microbiota composition and predicted functional capacity. **(A)** LEfSe analysis identifying discriminative taxa across sampling niches and groups; left, LDA scores (log10) for taxa enriched in each niche and condition, with colors indicating sample type and group (anastomosis, colonic content, and cecal content in CON and LPS), and right, a cladogram showing the phylogenetic distribution of LEfSe-identified biomarkers (LDA threshold as defined in Methods). **(B)** Longitudinal relative abundance of representative genera at the anastomotic site (Akkermansia, Mucispirillum, and Enterococcus) across POD 0–14 in the CON and LPS groups; data are shown as mean ± SEM. **(C)** Heatmap of *z*-score–normalized relative abundance for selected genera at the anastomotic site across POD 0–14 (columns) for the CON and LPS groups, with hierarchical clustering of taxa (rows). **(D)** PICRUSt2-predicted functional profiles at the anastomotic site displayed as a row-wise *z*-score heatmap across POD 0–14 for the CON and LPS groups; pathway labels highlighted in red denote functions related to bacterial motility/host interaction (epithelial invasion, chemotaxis, flagellar assembly, and two-component system), whereas labels highlighted in green denote repair-supportive metabolic functions (butanoate and glutathione metabolism, and starch/sucrose metabolism as a fermentable substrate–related pathway); color highlighting is for emphasis and does not indicate statistical significance. Statistical annotations: *p* < 0.05, *p* < 0.01, *p* < 0.001 for CON vs. LPS at the indicated time points (multiple-comparison procedure as described in Methods). LEfSe and PICRUSt2 analyses are exploratory, and PICRUSt2 denotes predicted functional potential rather than measured activity.

The heatmap further resolved the longitudinal clustering patterns (Figure 3C). In the LPS group, a prominent “pathobiont cluster” emerged during PODs 1–5 and was characterized by higher relative abundances of Enterococcus, Escherichia–Shigella, Klebsiella, and Proteus and lower relative abundances of beneficial taxa such as Akkermansia, Dubosiella, and Lactobacillus. A late-stage increase in the abundance of Mucispirillum was also observed. PICRUSt2 suggested differences in predicted functional potential for pathways related to bacterial invasion, chemotaxis, and flagellar assembly in the LPS group (Figure 3D), whereas the CON group showed greater representation of pathways related to butanoate and glutathione metabolism; these predictions were not direct measurements of metabolic activity. Collectively, these findings prompted us to test whether a fibre-based intervention was associated with microbial and metabolic changes and improved anastomotic repair.

### 3.3 LPS-associated impaired anastomotic transcriptomic programs governing the transition from inflammation to regeneration

We performed repeated-cross-sectional transcriptomic profiling of 41 independent anastomotic-tissue samples (3–5 mice per group–time cell) on PODs 0, 1, 3, 5, and 7 to define molecular programs associated with LPS-associated impaired anastomotic healing. Principal component analysis (PCA) revealed early separation of transcriptomes between groups, with maximal divergence at POD 5 (Figure 4A). Although transcriptomes of surviving mice showed partial convergence by POD 7, the number of DEGs between CON and LPS increased sharply towards POD 5 (Figure 4B), and heatmaps of the top 200 DEGs showed clear time-dependent clustering (Figure 4C). Later time points represent animals surviving to scheduled collection and may therefore be affected by survivor bias.

**Figure 4:**
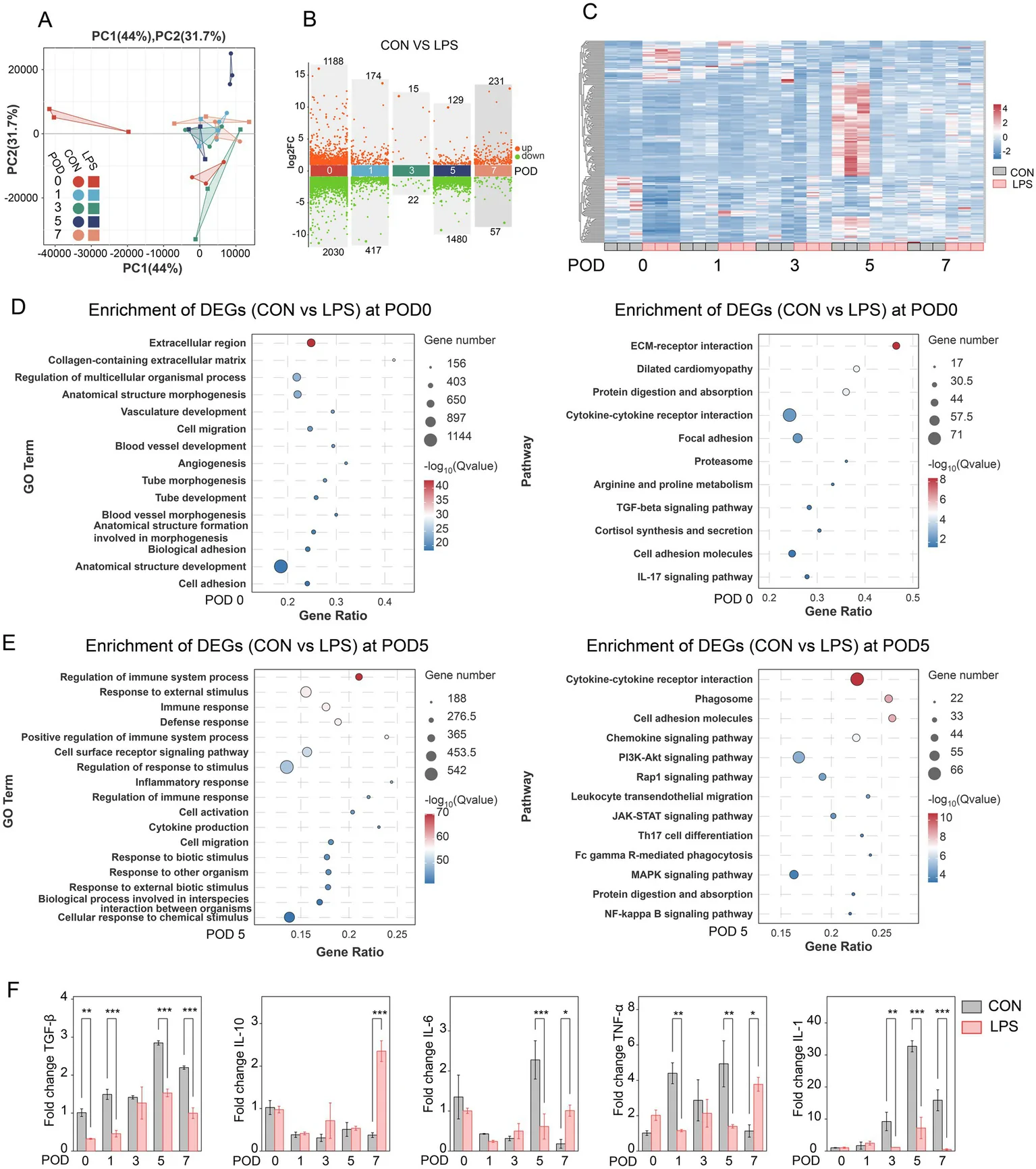
LPS priming disrupts anastomotic transcriptomic programs during the transition from inflammation to regeneration. **(A)** Principal component analysis (PCA) of anastomotic transcriptomes collected at POD 0, 1, 3, 5, and 7 from the CON and LPS groups. **(B)** Numbers of differentially expressed genes (DEGs) between CON and LPS at each POD; differential expression analysis was performed using DESeq2. Genes with |log2FC|>1 and Benjamini–Hochberg adjusted P (FDR) < 0.05 were defined as DEGs, with upregulated and downregulated genes shown separately. **(C)** Heatmap of the top 200 DEGs across groups and time points; expression values are row-normalized (*z*-score per gene) to visualize relative expression patterns, with the greatest separation observed at POD 5. **(D,E)** GO term enrichment (left) and KEGG pathway enrichment (right) of all DEGs at POD 0 **(D)** and POD 5 **(E)**; For KEGG enrichment, DEGs were mapped was assessed using a hypergeometric test against the whole-genome background with Benjamini–Hochberg correction. Bubble plots show gene ratios, with color indicating −log10(q value) and bubble size indicating the number of DEGs per GO term or pathway. **(F)** qPCR validation of representative genes across PODs; statistics were assessed by two-way ANOVA (group and POD) with *post hoc* multiple-comparisons testing. Data are presented as mean ± SEM. *p* < 0.05, *p* < 0.01, *p* < 0.001.

At POD 0, GO/KEGG enrichment analyses showed that DEGs in the CON group were enriched in categories including cell adhesion, collagen-containing ECM organization, and cell migration, with enrichment in ECM–receptor interaction and TGF-β signaling (Figure 4D). In contrast, DEGs in LPS-primed tissues were enriched in cytokine–cytokine receptor interaction pathways, indicating an early difference from the typical repair trajectory.

By POD 5, these early deviations progressed to a sustained imbalance in inflammation and repair pathways. Hierarchical clustering suggested that while the tissue formation programs of the CON group peaked, those of the LPS group remained in a pathology-associated state. The KEGG analysis indicated the enrichment of DEGs in leukocyte-trafficking and inflammatory-signaling categories (e.g., JAK–STAT and MAPK signaling) and lower representation of PI3K–Akt- and cell-adhesion-associated expression programs (Figure 4E). qPCR validation further confirmed a dysregulated cytokine pattern characterized by a blunted early inflammatory response (PODs 0–5) followed by pathological late-stage rebound (POD 7). Specifically, the levels of IL-6, TNF-α, and IL-1 were lower in the LPS group during the initial phase but exceeded the levels in the control group by POD 7, reflecting impaired inflammation resolution. Furthermore, the level of IL-10 increased on POD 7, suggesting a compensatory anti-inflammatory response, whereas reduced TGF-β signalling aligned with limited regenerative progression. Two-way ANOVA revealed significant group-by-time interactions for the levels of key cytokines (Figure 4F). Together, these findings indicate coordinated alterations in microbial ecology and host repair-related transcriptomic programs following LPS priming, accompanied by lower representation of PI3K–Akt-associated and cell-adhesion programs during the early postoperative window.

### 3.4 LPS-induced inflammatory priming is associated with impaired matrix maturation and altered inflammatory- and repair-associated macrophage-marker profiles

We quantified matrix deposition and local immune phenotypes to elucidate the histological remodeling and immune cell dynamics underlying mechanical vulnerability. Masson’s trichrome staining showed that LPS priming markedly impaired tissue remodeling, with reduced and disorganized collagen deposition at the anastomotic site in the LPS group (*p* < 0.001; Figures 5A,C). Consistent with this defective remodeling, the protein analysis revealed reduced TGF-β protein levels in endotoxaemic animals (*p* < 0.01; Figures 5G,I), which is consistent with impaired matrix maturation and limited reinforcement of the wound scaffold.

**Figure 5:**
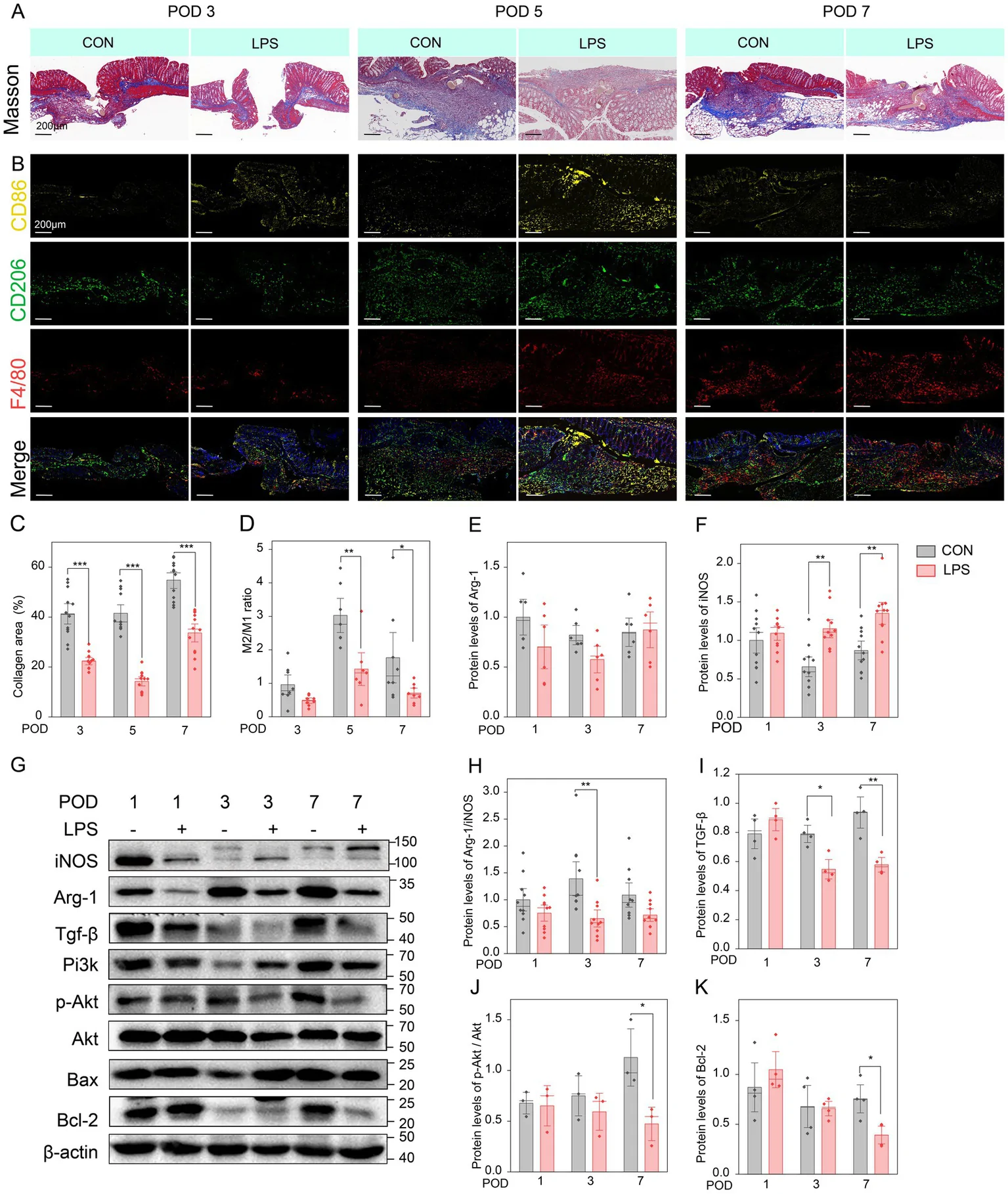
Endotoxemic stress impairs matrix maturation and is associated with altered inflammatory- and repair-associated marker profiles at the anastomosis. **(A)** Representative Masson’s trichrome staining of colonic anastomoses at POD 3, POD 5, and POD 7 in control (CON) and LPS-treated (LPS) mice; scale bar, 200 μm. **(B)** Representative immunofluorescence images of the anastomotic region stained for CD86, CD206, and F4/80 at POD 3, POD 5, and POD 7 (merged images include DAPI); scale bar, 200 μm. **(C)** Quantification of collagen-positive area from Masson’s staining. **(D)** CD206/CD86 marker ratio calculated as CD206^+^F4/80^+^/CD86^+^F4/80^+^ cell counts within the anastomotic region. **(E,F)** Densitometric quantification of Arg-1 **(E)** and iNOS **(F)** protein levels in anastomotic tissue. **(G)** Representative immunoblots for iNOS, Arg-1, TGF-β, PI3K, p-Akt, Akt, Bax, Bcl-2, and β-actin from anastomotic tissue at POD 1, POD 3, and POD 7. **(H–K)** Densitometric quantification of Arg-1/iNOS (H), TGF-β **(I)**, p-Akt/Akt ratio **(J)**, and Bcl-2 **(K)**; all targets were normalized to β-actin except p-Akt, which was normalized to total Akt. Data are presented as mean ± SEM with individual animals shown; each dot represents one mouse (*n* = 4–6 mice per group per time point). Statistical significance was assessed by two-way ANOVA (group × POD) followed by Tukey’s multiple-comparisons test. *p* < 0.05, *p* < 0.01, *p* < 0.001; ns, not significant.

This remodeling impairment occurred alongside an inflammation-dominant immune landscape. Multiplex immunofluorescence staining (PODs 3, 5, and 7) and protein analyses (PODs 1, 3, and 7) indicated a sustained proinflammatory shift at the anastomosis. In the LPS group, F4/80-associated and CD86-associated inflammatory markers were more prominent, accompanied by elevated iNOS expression (Figures 5B,D–F,H). In contrast, the CON group exhibited a higher CD206-associated repair-marker signal and lower iNOS expression. Accordingly, the CD206-to-CD86 marker ratio during the critical repair window was markedly reduced in LPS-primed animals, indicating a less repair-associated marker profile. In parallel, the p-Akt/Akt ratio was lower in the LPS group (*p* < 0.01; Figures 5G,J), accompanied by decreased Bcl-2 expression (*p* < 0.01; Figures 5G,K). Collectively, the lower TGF-β and p-Akt/Akt measurements and the inflammatory-associated marker profile were associated with the fragile tissue architecture and reduced mechanical strength observed in mice with endotoxaemia.

### 3.5 Postoperative inulin-supplemented EEN is associated with improved systemic recovery and greater anastomotic mechanical integrity in mice with endotoxaemia

We implemented postoperative EEN with or without inulin supplementation to examine whether the intervention was associated with improved recovery (Figure 6A). In the dedicated *n* = 10/group cohort followed through POD5, the global survival comparison was significant (log-rank χ^2^(3) = 7.820, *p* = 0.0499), but the planned LPS-DF versus LPS contrast was not significant after Holm correction (adjusted *p* = 0.2228; HR = 0.162, 95% CI 0.019–1.391); therefore, no specific survival-benefit claim is made. Full survival data, including event and number-at-risk values, are provided in Supplementary Table S8. The body-weight model showed an LPS × inulin × time interaction (χ^2^(5) = 82.998, *p* < 0.0001), with a POD5 LPS-DF versus LPS difference of 26.44 percentage points (95% CI 22.39–30.49; Holm-adjusted *p* < 0.0001). The average inulin effect on Murine Sepsis Score was −0.564 points (95% CI −0.923 to −0.206, *p* = 0.0020), although the three-way interaction and POD5 planned contrast were not significant. Evaluable n at POD5 was 9, 9, 5, and 9; survivor-only sensitivity analyses were directionally similar but are interpreted cautiously. Full mixed-effects and sensitivity-analysis results are provided in Supplementary Table S9.

**Figure 6:**
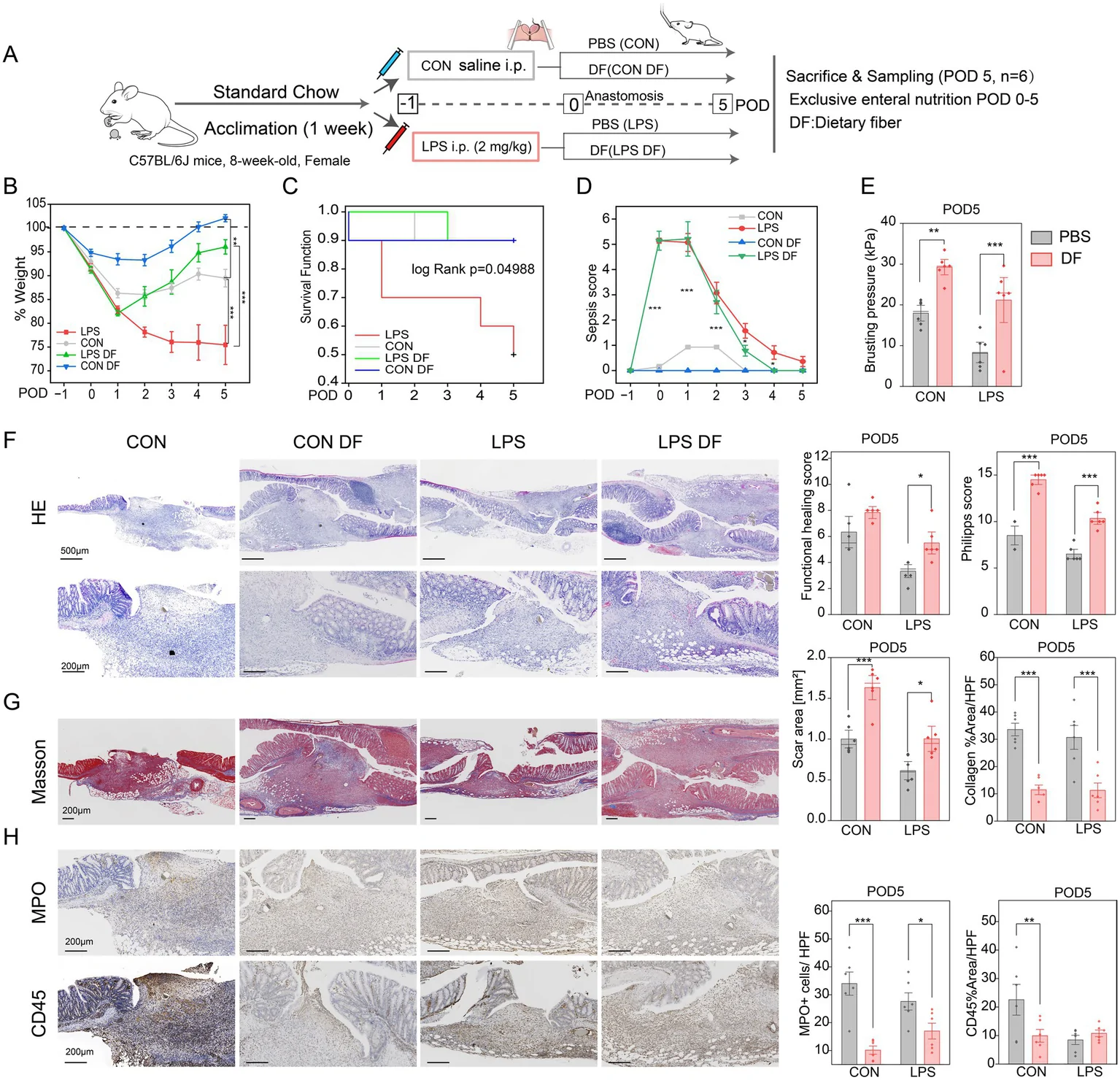
Experiment 2: postoperative inulin-supplemented EEN and early anastomotic healing under LPS priming. **(A)** Experimental design. A dedicated survival/longitudinal cohort included *n* = 10/group and was followed through POD5; an independent terminal cohort provided six POD5 survivors/group for ABP, histology, and immunohistochemistry. **(B)** Body-weight change through POD5. **(C)** Kaplan–Meier survival curves through POD5 with number at risk; events were 1, 1, 5, and 1 in CON, CON-DF, LPS, and LPS-DF. Death or attainment of a predefined humane endpoint was treated as an event; animals alive at POD5 were administratively censored. Full survival data and number-at-risk values are provided in Supplementary Table S8. **(D)** Murine Sepsis Scores. **(E)** ABP (kPa) at POD5. **(F)** H&E and healing scores. **(G)** Masson staining, scar/granulation area (mm^2^), and collagen-positive fraction. **(H)** MPO and CD45 staining. Five fields were averaged within each mouse, and each plotted point represents one mouse (*n* = 6/group for **E–H**). **(B)** and **(D)** were analyzed using mixed-effects models (full outputs in Supplementary Table S9); POD5 outcomes used 2 × 2 factorial models with Tukey-adjusted comparisons. Data are mean ± SEM with individual animals shown.

We next assessed POD5 anastomotic mechanical integrity. In the factorial model, LPS lowered ABP by 8.86 kPa (95% CI −13.48 to −4.25; *F*(1,20) = 16.06, *p* = 0.0007), whereas inulin supplementation increased ABP by 12.08 kPa (95% CI 7.47–16.70; *F*(1,20) = 29.84, *p* < 0.0001); the interaction was not significant. H&E findings and healing scores were consistent with improved POD5 tissue repair (Figure 6F). Inulin increased the scar/granulation area (mm^2^) but reduced the collagen-positive fraction within the ROI (Figure 6G); thus, improved ABP occurred despite lower collagen density, and the structural mechanism cannot be inferred without later time points or collagen-composition/organization measurements. MPO and CD45 staining were lower with inulin supplementation (Figure 6H). Full factorial estimates and interactions are reported in Supplementary Table S6.

### 3.6 Postoperative inulin-supplemented EEN is associated with cell-survival measures and inflammatory- and repair-associated immune markers

We characterized cellular changes accompanying the improvement in anastomotic repair following inulin-supplemented EEN by evaluating macrophage-associated marker profiles, epithelial turnover, and apoptosis in the anastomotic granulation tissue. Quantification was performed on one section per mouse, with five randomly selected fields per section and the ROI defined as granulation tissue at the anastomotic site. The five fields were averaged within each mouse before analysis, and each plotted point represents one mouse.

Multiplex immunofluorescence showed treatment-associated changes in the local marker profile. Inulin had a main effect on the CD206^+^/CD86^+^ ratio (*p* = 0.0167), whereas the interaction was not significant (*p* = 0.0767); the Tukey-adjusted LPS-DF versus LPS comparison was significant (*p* = 0.023). These markers are described as inflammatory- and repair-associated profiles rather than discrete M1/M2 polarization states.

Ki67 staining showed an inulin main effect (*p* = 0.0008; Figures 7C–D). TUNEL positivity showed an LPS × inulin interaction (*p* = 0.021), and the Tukey-adjusted LPS-DF versus LPS comparison was significant (*p* = 0.0065; Figures 7E–F). These findings indicate concurrent differences in proliferation- and apoptosis-associated measures at POD5.

**Figure 7:**
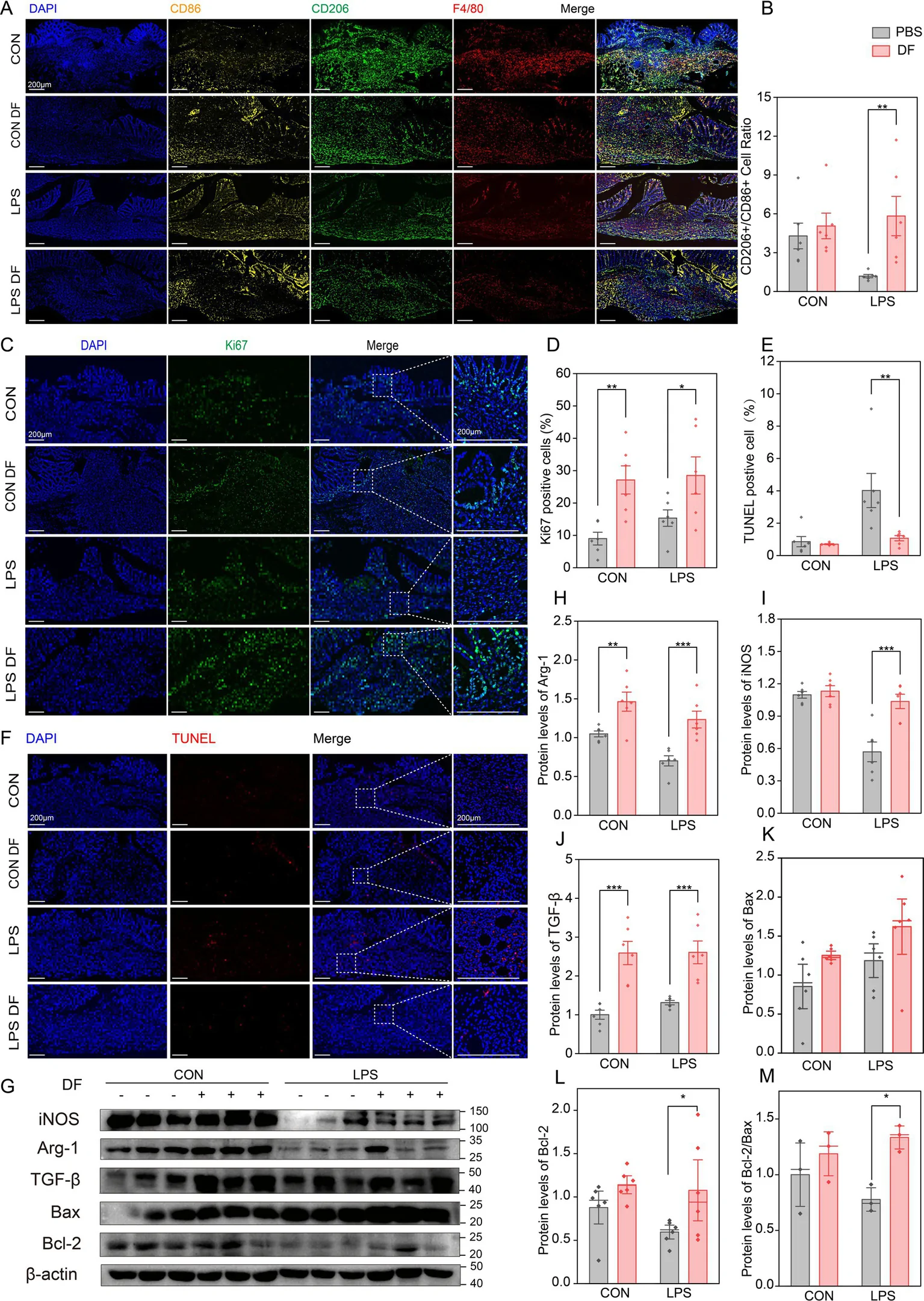
Postoperative inulin-supplemented EEN and cell/immune-marker measures in POD5 anastomotic granulation tissue. **(A–F)** CD86, CD206, F4/80, Ki67, and TUNEL staining with quantification. One section per mouse and five non-overlapping fields per section were analyzed; fields were averaged within mouse, each point represents one mouse, and *n* = 6/group. **(G–L)** Whole-tissue immunoblots and densitometry for iNOS, Arg-1, TGF-β, Bax, and Bcl-2. M, Bcl-2/Bax ratio from three different mice/group (n = 3/group); the ratio was analyzed on the natural-log scale. POD5 outcomes were analyzed using 2 × 2 factorial models, with multiplicity-adjusted simple-effect comparisons where interactions were detected; full results are in Supplementary Table S6. iNOS was higher in LPS-DF than LPS (Holm-adjusted *p* = 0.00014) and is interpreted as a whole-tissue marker difference rather than evidence of macrophage polarization. Data are mean ± SEM. Scale bars are indicated in the panels.

Whole-tissue Western blotting showed treatment-associated differences in Arg-1 and TGF-β (Figures 7G–J); these signals were not assigned specifically to macrophages. iNOS abundance showed an LPS × inulin interaction (*F*(1,20) = 11.52, *p* = 0.0029, partial η^2^ = 0.365). Inulin had no detectable effect in the CON background (Holm-adjusted *p* = 0.711) but increased iNOS abundance in LPS-primed mice (estimated difference, 0.468; 95% CI, 0.279–0.656; Holm-adjusted *p* = 0.00014). Because iNOS was measured in whole anastomotic tissue, this is interpreted as a tissue-level marker difference rather than evidence of macrophage polarization. The Bcl-2/Bax ratio was calculated from three different mice/group. In the prespecified natural-log ratio model, no effect of LPS (*p* = 0.447), inulin (*p* = 0.990), or their interaction (*p* = 0.810) was detected; therefore, no ratio-based anti-apoptotic claim is made.

### 3.7 Postoperative inulin-supplemented EEN is associated with microbial-community and short-chain-fatty-acid differences

We performed 16S rRNA sequencing to determine whether microbiome changes accompanied the healing outcomes. PCoA showed differences in community structure across groups (PERMANOVA *R*^2^ = 0.4051, *p* = 0.001; Figure 8D), although this analysis does not establish causality. For Shannon diversity (CON *n* = 7, CON-DF *n* = 4, LPS *n* = 8, LPS-DF *n* = 8), inulin had a negative main effect (estimate −1.129, 95% CI −1.693 to −0.566; *F*(1,23) = 17.18, *p* = 0.00039); neither the LPS main effect nor the interaction was significant. All 27 available POD5 samples were included; no samples were excluded after sequencing or during statistical analysis.

**Figure 8:**
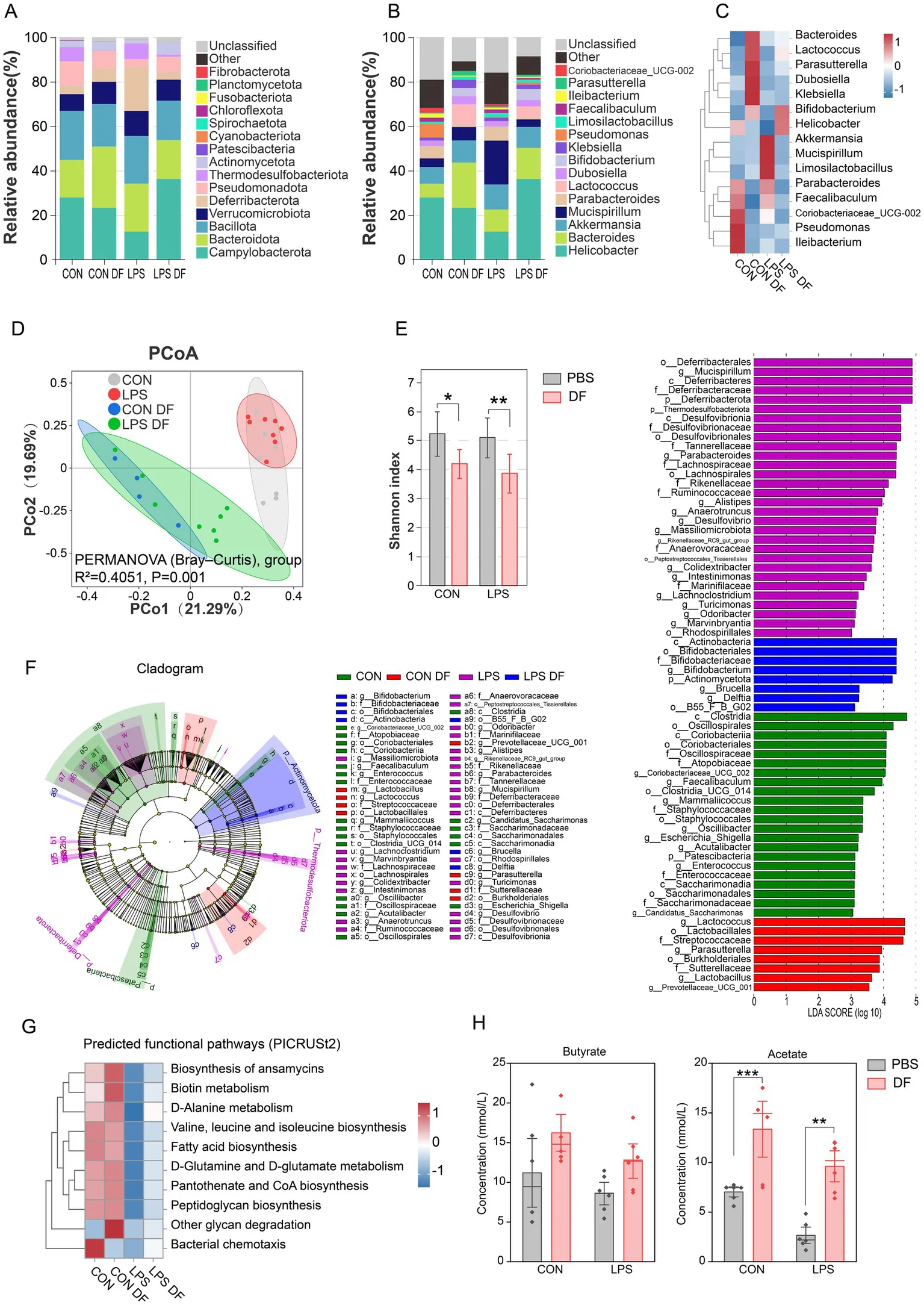
POD5 microbiome and short-chain-fatty-acid findings. **(A,B)** Phylum- and genus-level relative abundances. **(C)** Row-wise z-score heatmap. **(D)** Bray–Curtis PCoA with PERMANOVA. **(E)** Shannon diversity (CON *n* = 7, CON-DF *n* = 4, LPS *n* = 8, LPS-DF *n* = 8); all 27 available POD5 samples were included, with no exclusions after sequencing or during statistical analysis. **(F)** Exploratory LEfSe findings. **(G)** PICRUSt2-predicted functional potential. **(H)** Colonic-content butyrate and acetate concentrations by HPLC (mmol/L; *n* = 6/group). E and H were analyzed using 2 × 2 factorial models; full main effects/interactions and Tukey-adjusted pairwise comparisons are in Supplementary Table S7. **(H)** Annotation denotes the acetate LPS-DF versus LPS comparison (Tukey-adjusted *p* = 0.0015); the corresponding butyrate comparison was not significant (*p* = 0.421). Each point represents one mouse. Data are mean ± SEM.

Taxonomic profiles showed treatment-associated differences in relative abundance (Figures 8A–C,F); because the data are compositional and no absolute bacterial-load measurement was performed, these findings are not described as true expansion or depletion. PICRUSt2 results are interpreted only as predicted functional potential (Figure 8G). For acetate (*n* = 6/group), LPS had a negative main effect (−4.06, *p* = 0.0017) and inulin had a positive main effect (+6.64, *p* < 0.0001), with no interaction; LPS-DF exceeded LPS after Tukey adjustment (+6.96, 95% CI 2.51–11.40, *p* = 0.0015). For butyrate, inulin had a positive main effect (+4.56, *p* = 0.0228), whereas the LPS-DF versus LPS comparison was not significant (*p* = 0.421). These colonic-content measurements accompany the healing outcomes but do not establish mediation.

## 4 Discussion

AL remains a formidable challenge in colorectal surgery, especially in patients who present with preoperative peritonitis or endotoxaemia secondary to obstruction (Bakker et al., 2014). Our findings suggest that endotoxaemia is associated with a localized pathological state in which microbial alterations and host repair processes appear maladaptively coupled during the early postoperative window (Alam et al., 2016). This co-occurrence was more evident within the anastomotic tissue itself than in the luminal areas, supporting the idea that mucosa-associated microbes and their spatial distribution are crucial for anastomotic healing (Hyoju et al., 2020; Bakker et al., 2014; Hajjar et al., 2023; Zhang et al., 2025; Leonardi et al., 2022). The observed compromise in mechanical integrity and increased likelihood of leaks under endotoxemic stress underscore the extreme vulnerability of this microenvironment.

A significant insight from our microbiome analysis is the niche-specific nature of microbial succession during the postoperative period. Rather than inducing uniform alterations across the gastrointestinal tract, endotoxaemia selectively perturbs the anastomotic niche during the initial phase of repair (van Praagh et al., 2019; van Praagh et al., 2016). An opportunism-enriched microbial pattern emerges shortly after surgery and persists throughout the remodeling phase. This ecological shift involves lower relative abundance of mucoprotective taxa and higher relative abundance of pathobionts, consistent with an association between microbial composition and healing at the wound site. Notably, our imaging data provide spatial context: the anastomoses of mice with endotoxaemia exhibit focal bacterial aggregates along sutures and damaged surfaces, without a higher relative EUB338-to-DAPI ratio (Baumgartner et al., 2021). These observations support the possibility that microspatial organization, in addition to composition, may influence host–microbe interactions during early repair (Leonardi et al., 2022). While microbiota-mediated causality remains to be tested using fecal microbiota transplantation (FMT), our data suggest a spatial association between dysbiosis and matrix breakdown at the anastomotic site (Shogan et al., 2015; Kandhwal et al., 2022; Jacobson et al., 2020). Prior work suggests that opportunistic bacteria can contribute to collagen degradation at the wound margin via microbe-derived proteases and host inflammatory responses (Shi et al., 2022; Alverdy and Schardey, 2021). Specific pathogens can also activate host plasminogen at the anastomotic site (Jacobson et al., 2021; Jacobson et al., 2020). Together, these observations are consistent with an association between pathobiont enrichment, focal microbial aggregation, and impaired matrix reinforcement; direct tests of strain-level or proteolytic causality are required.

At the host transcriptional level, endotoxaemia is associated with a failure to engage in timely reparative programs during an outcome-critical early window of anastomotic healing (Morgan and Shogan, 2022; Qiu et al., 2024). In controls, gene expression rapidly shifted towards wound initiation and matrix organization, whereas endotoxaemia biased the anastomotic tissue towards inflammatory receptor signalling (Sommer et al., 2013; van Praagh et al., 2021). During the remodeling phase, the anastomoses of mice with endotoxaemia showed sustained leukocyte trafficking together with lower representation of expression programs related to cell survival, adhesion, and tissue reconstruction, most notably the PI3K–Akt and TGF-β pathways (Landén et al., 2016; Karrasch et al., 2011; El-Assal and Besner, 2005; Beck et al., 2003; Morgan and Shogan, 2022). The accompanying cytokine dynamics were consistent with impaired inflammation resolution and an incomplete transition from acute inflammation to repair. In parallel, immune and histological analyses provided a cellular basis for this impaired transition: anastomoses of mice with endotoxaemia displayed a marker profile with greater inflammatory-associated and lower repair-associated signals (Sommer et al., 2013; Strowitzki et al., 2021). These immune-marker differences were accompanied by lower p-Akt/Akt measurements and an increase in apoptosis. While our current evidence is descriptive and does not include the pharmacological perturbation of Akt signaling, the alignment between transcriptomic patterns and protein-level readouts is consistent with the altered engagement of repair-related pathways following LPS priming. Future studies using pathway-level perturbations will be needed to establish causality.

A significant contribution of this study is the observation that a short postoperative inulin-supplemented EEN regimen was associated with better systemic recovery and anastomotic strength. At the tissue and cellular levels, the intervention was accompanied by lower inflammatory infiltration, higher proliferative activity, lower TUNEL positivity, and changes in inflammatory- and repair-associated markers. Inulin increased the scar/granulation area but reduced the collagen-positive fraction at POD5; therefore, the structural mechanism underlying the higher ABP remains unresolved. In parallel, the intervention changed microbial relative-abundance profiles and was associated with an altered colonic-content SCFA profile, including higher acetate. Prior studies support the biological plausibility of diet–microbiota–healing relationships and potential SCFA effects (Boatman et al., 2023; Bosmans et al., 2016). However, the present study did not test mediation, and the LPS-DF versus LPS butyrate comparison was not significant after adjustment. Antibiotic depletion, microbiota transfer, direct metabolite supplementation, receptor blockade, or formal mediation experiments would be required to determine whether microbiota or individual SCFAs caused the healing effect.

These findings offer promising directions for translational research, particularly in intervention strategies. The observed association with a targeted, brief postoperative inulin-supplemented EEN course supports further evaluation of its clinical practicality, especially in scenarios where preoperative optimization is limited or not feasible. This early “postoperative intervention window” may warrant evaluation in appropriately selected patients. Clinically, however, the implementation of fibre-enriched nutrition must be balanced against individual tolerance. Our findings should be viewed as hypothesis-generating proof-of-concept; any clinical application would require prospective testing and should be limited to hemodynamically stable patients with returning bowel function. Moreover, enhanced recovery after surgery (ERAS) pathways prioritize early mobilization and early advancement of oral/enteral intake to support gastrointestinal recovery (Gustafsson et al., 2025). Any postoperative DF strategy should use cautious dosing, attention to fibre type, and close tolerance monitoring (Weimann et al., 2025).

Several limitations of this study warrant consideration. While the LPS challenge provides a standardized model of acute inflammatory priming, it does not fully reproduce clinical polymicrobial sepsis or other contributors to anastomotic leakage, including obstruction, ischemia, fecal contamination, malnutrition, inflammatory bowel disease, and neoadjuvant treatment. Formula intake was not recorded; therefore, individual inulin, energy, and nutrient exposure could not be quantified, and pair-feeding and viscosity- or osmolality-matched controls were not included. POD5 terminal outcomes were evaluated in animals surviving to scheduled collection and may therefore be subject to survivorship bias. Although 16S rRNA-based functional inference is useful for generating hypotheses, the original 97%-OTU workflow did not include extraction blanks, PCR-negative controls, dedicated contaminant filtering, absolute bacterial-load measurements, preintervention microbiota assessment, litter-effect control, cage-aware modeling, ASV reconstruction, or compositional differential-abundance analysis. Accordingly, findings from low-biomass tissue samples and exploratory LEfSe and PICRUSt2 analyses should be interpreted cautiously, and FISH/SEM demonstrate bacterial localization or aggregation rather than definitive biofilm formation. SCFA measurements were limited to colonic contents and do not establish exposure within anastomotic tissue or portal circulation. HPLC quantification used an external-standard method without an internal standard, and archived documentation did not contain complete information on recovery, calibration range/linearity, LOD/LOQ, or chromatographic specificity. RNA quality was assessed using NanoDrop spectrophotometry and gel electrophoresis, but numerical RIN values were not included in the retained reports. Complete cross-assay linkage to individual Mouse IDs was not available for all sequencing samples, precluding individual-level multi-omics integration. The exclusive use of female mice may also limit generalizability across sexes. Finally, the study identified concurrent associations among nutritional intervention, microbial features, SCFA profiles, and healing outcomes but was not designed to establish microbiota- or SCFA-mediated causality. Future studies should validate these findings in more clinically representative models and integrate higher-resolution metagenomic, metabolomic, and spatial approaches (Matchado et al., 2024; Dejager et al., 2011).

In summary, LPS-induced inflammatory priming was associated with impaired anastomotic repair, niche-specific microbial changes, and altered host repair programs. Postoperative inulin-supplemented EEN improved several early healing outcomes and was accompanied by microbial-community and SCFA differences. These concurrent findings support further study of perioperative nutritional strategies but do not establish that microbiota remodeling or SCFAs mediated the healing effects; validation in more clinically representative models is required.

## Data Availability

The raw sequencing data are publicly available in the Genome Sequence Archive (GSA) under BioProject PRJCA058182. The RNA-seq data are available under accession CRA038770 (https://ngdc.cncb.ac.cn/gsa/browse/CRA038770). The 16S rRNA gene sequencing data are available under accessions CRA038882 (https://ngdc.cncb.ac.cn/gsa/browse/CRA038882) and CRA049295 (https://ngdc.cncb.ac.cn/gsa/browse/CRA049295). Processed feature/count matrices, sample metadata, figure source data, and analysis code are available in Zenodo at https://doi.org/10.5281/zenodo.22272055.
